# The Challenging Aspect of Macrophage Activation Syndrome in the Setting of Sepsis or Systemic Inflammatory Response Syndrome (SIRS)

**DOI:** 10.7759/cureus.36228

**Published:** 2023-03-16

**Authors:** Said Benlamkaddem, Djoudline Doughmi, Imane Tlamçani, Mohamed Adnane Berdai, Mustapha Harandou

**Affiliations:** 1 Maternal and Pediatric Critical Care Unit, Hassan II University Hospital, Fez, MAR; 2 Faculty of Medicine and Pharmacy, Sidi Mohamed Ben Abdellah University, Fez, MAR; 3 Hematology Laboratory, Hassan II University Hospital, Fez, MAR

**Keywords:** thrombocytopenia, bone marrow puncture, hemophagocytosis, sirs, sepsis, macrophage activation syndrome

## Abstract

Macrophage activation syndrome (MAS) is a rare but potentially fatal disease. It is characterized by hyperinflammation, including the proliferation and activation of immune cells (CD8 T cells and NK cells) associated with hypercytokinemia. Patients present with fever, splenomegaly, and cytopenia, associated with a hemophagocytosis picture in the bone marrow. It can progress to a multiorgan failure syndrome (MODS), mimicking sepsis or a systemic inflammatory response syndrome (SIRS).

We report the case of an 8-year-old girl admitted to the pediatric intensive care unit for the management of major trauma due to a domestic accident. She presented with a protracted fever in the context of a septic shock, despite appropriate treatment. The association with bicytopenia, hyperferritinemia, hypofibrinogenemia, and hypertriglyceridemia was suggestive of MAS which was confirmed by a bone marrow puncture showing hemophagocytosis. A Bolus of corticotherapy was then added to the supportive treatment and broad-spectrum antibiotherapy, with a good outcome.

## Introduction

Macrophage activation syndrome (MAS) is a rare but potentially fatal disease. It is defined by clinical signs like fever and splenomegaly, biological abnormalities including cytopenia, low fibrinogen, high plasma triglycerides, and hyperferritinemia, associated with a hemophagocytosis picture in the bone marrow [1]. The condition arises due to a disorder (overactivation and/or defect) of the immune system (CD8 T cells and natural killer (NK) cells) causing a cytokine storm [2], which can progress to multiple organ dysfunction syndrome (MODS) mimicking sepsis or a systemic inflammatory response syndrome (SIRS).

We report the case of an 8-year-old girl who was admitted to the pediatric intensive care unit for the management of major trauma due to a domestic accident. She was diagnosed with MAS in the context of SIRS (major trauma) and septic shock due to ventilator-acquired pneumonia.

This case report emphasizes the importance of a systematic approach for distinguishing between the three syndromes: MAS, sepsis, and SIRS.

## Case presentation

An 8-year-old girl, with no clinical history, was brought to the emergency department for management of major trauma after a fall from the 5th floor. The first clinical examination showed a fully conscious, oriented patient with a regular pulse rate of 150 beats/min (BPM), blood pressure at 70/50  mmHg, cold extremities, polypnea at 40 breaths/min, and oxygen saturation at 80% in room air.

After stabilization, a body CT scan was performed which showed a grade 2 thoracic aortic injury (intramural hematoma) located at the isthmus, a large pneumothorax, pneumomediastinum, pneumopericardium, bilateral pulmonary contusion, splenic and hepatic laceration, active bleeding from gluteal arteries, and multiple broken bones. She underwent chest drainage and was then taken to the arteriography room, where an embolization of the hypogastric arteries was performed. She exhibited ventricular fibrillation due to multiple myocardial contusions (troponin level at 40.538 ng/ml) and recovered after cardiac massage and defibrillation.

During her hospitalization, the patient was initially ventilated and sedated, afebrile, and underwent multiple routine blood tests that were generally normal. After weaning her off sedation, she had a delayed awakening. A control brain CT scan was then performed, revealing multiple ischemic areas.

Within 3 weeks, she developed a protracted fever. It was first attributed to septic shock due to ventilator-associated pneumonia caused by *Acinetobacter baumanii*, with a white blood cell (WBC) level at 6 x 103 /µL, C reactive protein at 200 mg/L, serum lactate level at 4.5 mmol/L and vasopressor requirement to maintain a mean arterial pressure of 65 mmHg. The fever (40-41 °C) persisted despite the administration of broad-spectrum antibiotics such as colistin, meropenem, and teicoplanin, with a lactate level decreasing to less than 2 mmol/L, with no more requirement of norepinephrine.

A complete blood count was performed and showed bicytopenia (hemoglobin level at 4.8 g/dL and platelets count at 7 x 103 /µL), elevated biochemical markers including elevated ferritin at 4787 µg/l and triglycerides at 3.1 g/L, severe hypofibrinogenemia at 0, elevated aspartate and alanine aminotransferases (296, 114 UI/L) and lactate dehydrogenase (LDH) (1132 UI/L) (Table 1).

**Table 1:** Laboratory findings before and after corticosteroid therapy ASAT: aspartate aminotransferase; ALAT: alanine aminotransferase; LDH: lactate dehydrogenase

Parameters	Admission	Day 1 of corticosteroids	Day 3	Week 2	Week 4	Normal range
Hemoglobin (g/dL)	9.9	4.8	9.5	12	13	11.5-15
Platelets (x10^3^ /µL)	161	7	22	371	357	150-450
Ferritin (µg/l)	-	4787	-	1400	941	10-120
Triglyceride (g/l)	-	3.1	-	-	2.43	0-1.5
ASAT (UI/L)	410	296	92	37	32	0-35
ALAT(IU/L)	94	114	70	16	13	0-35
LDH (IU/L)	-	1132	-	-	240	0-240
Protein C reactive (mg/L)	6	206	150	48	29	< 5
Procalcitonin ng/mL)	-	2.28	-	0.27	0.1	< 0.5
Fibrinogen (g/L)	0	0	-	-	4.36	2.38-4.98

Once macrophage activation syndrome was suspected, the patient received a methylprednisolone bolus (500 mg/m2 of body surface area) and a transfusion of red blood cells and platelets.

**Figure 1:**
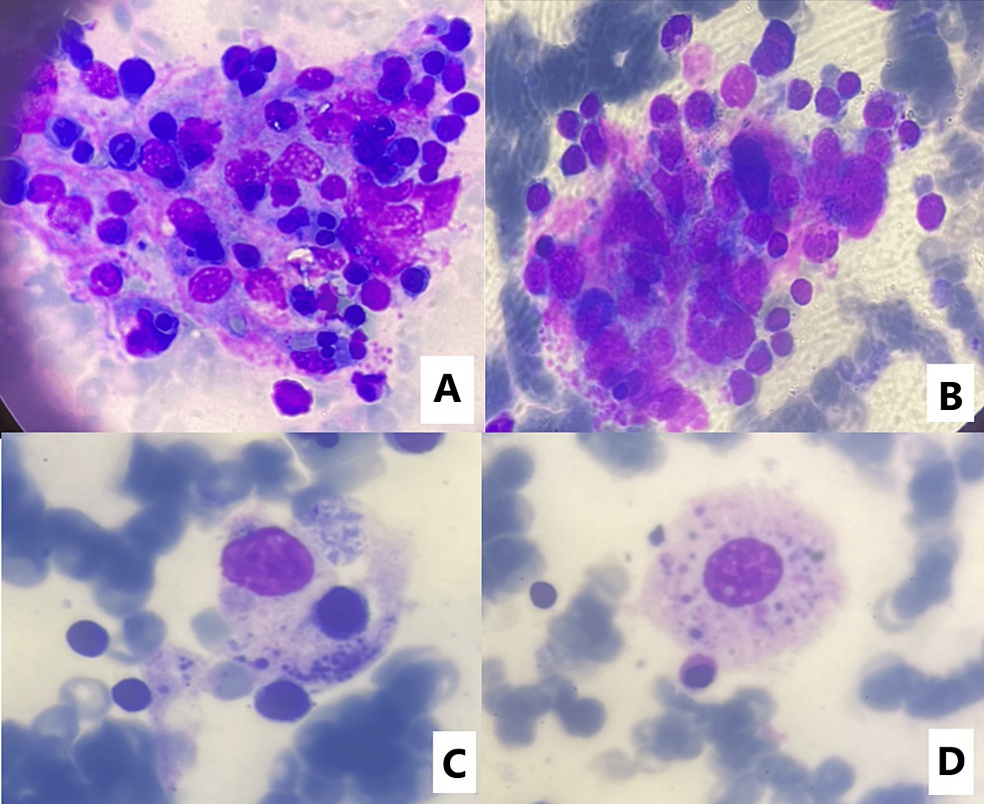
Hemophagocytic cells in bone marrow smear (MGG; Optical microscope x100). Macrophages phagocytosing mature red blood cells (RBC) and nucleated RBC precursors, neutrophils and granulocyte precursors, plasma cells, and lymphocytes (A-B). Macrophage, including a red blood cell and platelets (C). Example of a histocyte-ingested platelet that we do not consider to be a definite hemophagocyte (D). MGG: May-Grünwald Giemsa coloration

We completed the investigation by a bone marrow puncture at the iliac crest that revealed a normocellular and heterogeneous bone marrow containing many megakaryocytes with a normal morphological appearance. The marrow cytology smear revealed a normal and equilibrated marrow of both erythroblastic and granular lineages with the presence of all maturation states; 1% blasts were identified in the marrow. We also differentiated macrophages actively phagocytosing hematopoietic cells (Figure 1).

Based on the clinical, biological, and cytological criteria, the diagnosis of macrophage activation syndrome (MAS) was retained.

The evolution was favorable, as the patient became afebrile after 3 days of corticosteroid therapy; the control blood test showed an improvement of hemoglobin and platelets levels (12 g/dL, 371 x 103 /µL), with a decreasing level of ferritin (1400 µg/l), and aspartate and alanine aminotransferases (37 and 16 UI/L, respectively).

She was weaned from mechanical ventilation after nearly 2 months of ICU stay and is now undergoing rehabilitation.

## Discussion

Macrophage activation syndrome is characterized by an abnormal proliferation and activation of the monocyte-macrophage which causes blood cell phagocytosis [2-4]. Though the incidence of this pathology in the intensive care unit is between 0.8% and 4%, it seems to be underestimated [5,6].

The pathophysiology of macrophage activation syndrome is very complex and involves overactivation and overproduction of T cells and macrophages [2,7]. Extensive hypercytokinemia is associated strongly with its pathogenesis. The overproduction of interleukin (IL)-1, IL-6, IL-12, and IL-18, interferon (IFN)-g, and tumor necrosis factor (TNF)-α is closely associated with the pathogenesis of MAS [8]. Several factors are recognized as possible causes triggering MAS: infections, hematologic malignancies, systemic diseases, etc. [1,2,8].

The clinical presentation of MAS is usually acute and may sometimes be dramatic, with a rapid onset of a multiorgan failure that requires multidisciplinary medical care. Fever is the main clinical manifestation of MAS, related to the secretion of IL-1 and TNF-α. Almost all patients have an enlarged spleen [2,9].

Typical laboratory values are cytopenia (the consequence of phagocytosis of hematopoietic precursor), more frequent thrombocytopenia and anemia, and less frequent neutropenia. This cytopenia very often progresses to pancytopenia, noted in 75% of patients in the literature. Other biochemically characteristic findings are high triglycerides, low fibrinogen or global coagulation disorder, and high ferritin, which can be explained by hypercytokinemia (TNF-α, INF-g, IL-1, -2, -6, -8, -10, -12, -18, etc.)(Table 2) [10]. High transaminases, bilirubin and LDH, low total protein, and hyponatremia are less frequently found. A bone marrow examination is mandatory but only a minority of patients have hemophagocytosis pictures [3,11].

**Table 2:** The diagnostic criteria of macrophagic activation syndrome established by the Histiocyte Society. Five of the eight criteria are considered necessary for the diagnosis. NK cell: natural killer cell Source: Henter et al. [10]

Clinical criteria
Fever
Splenomegaly
Laboratory criteria
Cytopenia ≥ 2 cell lines
Hypertriglyceridemia (>3 mmol/L) and/or hypofibrinogenemia (≤1.5 g/L)
Ferritin ≥500 µg/L
sCD25 ≥2400 U/mL
Decreased or absent NK-cell activity
Cytological criteria
Hemophagocytosis in bone marrow, liver, spleen, or lymph nodes.

Based on these criteria, differentiating between MAS, sepsis, and SIRS is challenging because they share common features. Indeed, hypercytokinemia, which is the key to MAS pathogenesis, is also considered the cornerstone of sepsis and SIRS pathophysiology, leading to all the aforesaid clinical and biological abnormalities. Meanwhile, studies show that hemophagocytosis might be relatively common in critically ill patients without MAS, particularly those with sepsis and multiorgan failure (MODS) [12,13]. It has been reported in 12% of patients with SIRS [14] and 60% with severe sepsis and thrombocytopenia [5]. The immunodepression caused by SIRS and sepsis may promote infection, which can trigger MAS [15].

This case report perfectly illustrates this dilemma, as our patient had SIRS due to major trauma; she also developed a septic shock on *Acinetobacter baumanii *ventilator-acquired pneumonia [16]. The diagnosis of MAS was then discussed based on the persistence of fever and biological disorder, despite appropriate treatment for septic shock. This approach may be judicious as the MAS diagnosis criteria are, in our opinion, unable to distinguish between the three syndromes, and the early initiation of an immunosuppressive therapy could improve the outcome.

The medical management of MAS must be multidisciplinary, including supportive therapy (vasopressors, oxygen therapy, mechanical ventilation, transfusion, etc.) associated with a specific treatment of the triggering factor (anti-infectious treatment, chemotherapy, etc.) [12,13].

Currently, most MAS therapeutic protocols are derived from the treatment of primary (genetic) forms. Most studies recommend a combination of corticosteroids and systemic immunosuppression (etoposide, cyclosporin A, etc.) [1,8,9,11]. The effectiveness of IV immunoglobulin is not consistent [15].

It is probably more judicious for patients with sepsis or SIRS who develop the clinical picture of MAS to be placed on a specific treatment and supportive care. However, considering that all three syndromes represent severe hyperinflammatory states, it seems reasonable to use a short course of corticosteroids and/or IV immunoglobulin treatment (but not of cytostatic drugs) to control hypercytokinemia in patients who do not improve on supportive therapy or progress to organ failure [9].

Mortality is significant, ranging from 20% to 88%. In intensive care units, mortality is more than 50%, especially in patients with shock at admission or with severe thrombocytopenia [17]. The literature suggests that only a decrease in ferritin levels by more than 50% after treatment appears to be associated with reduced mortality [18].

## Conclusions

The issue raised by this case report needs further work and large studies to identify more diagnostic criteria to clarify adequately the difference between the three syndromes (MAS, sepsis, and SIRS) and to elaborate optimal treatment protocols to improve decision-making (immunosuppressive therapy versus sepsis) and outcomes.

## References

[1] Gonzalez F, Vincent F, Cohen Y (2009). Syndrome d’activation macrophagique d’origine infectieuse: étiologies et prise en charge [Infection-related hemophagocytic syndrome: aetiologies and management]. Réanimation.

[2] Shimizu M (2021). Macrophage activation syndrome in systemic juvenile idiopathic arthritis. Immunol Med.

[3] Allen CE, Yu X, Kozinetz CA, McClain KL (2008). Highly elevated ferritin levels and the diagnosis of hemophagocytic lymphohistiocytosis. Pediatr Blood Cancer.

[4] Rekik R, Morazin F, Lumbroso A, Stirnemann J, Montravers P, Gauzit R (2004). [Reactive haemophagocytic syndrome and multiple organ failure in intensive care unit patients]. Ann Fr Anesth Reanim.

[5] Stéphan F, Thiolière B, Verdy E, Tulliez M (1997). Role of hemophagocytic histiocytosis in the etiology of thrombocytopenia in patients with sepsis syndrome or septic shock. Clin Infect Dis.

[6] Strauss R, Neureiter D, Westenburger B, Wehler M, Kirchner T, Hahn EG (2004). Multifactorial risk analysis of bone marrow histiocytic hyperplasia with hemophagocytosis in critically ill medical patients--a postmortem clinicopathologic analysis. Crit Care Med.

[7] Créput C, Galicier L, Buyse S, Azoulay E (2008). Understanding organ dysfunction in hemophagocytic lymphohistiocytosis. Intensive Care Med.

[8] Chandrakasan S, Filipovich AH (2013). Hemophagocytic lymphohistiocytosis: advances in pathophysiology, diagnosis, and treatment. J Pediatr.

[9] Janka GE, Lehmberg K (2014). Hemophagocytic syndromes--an update. Blood Rev.

[10] Henter JI, Horne A, Aricó M (2007). HLH-2004: Diagnostic and therapeutic guidelines for hemophagocytic lymphohistiocytosis. Pediatr Blood Cancer.

[11] Roberts I (2006). Histiocytic disorders of children and adults: basic science, clinical features and therapy. Bone Marrow Transplant.

[12] Raschke RA, Garcia-Orr R (2011). Hemophagocytic lymphohistiocytosis: a potentially underrecognized association with systemic inflammatory response syndrome, severe sepsis, and septic shock in adults. Chest.

[13] Castillo L, Carcillo J (2009). Secondary hemophagocytic lymphohistiocytosis and severe sepsis/systemic inflammatory response syndrome/multiorgan dysfunction syndrome/macrophage activation syndrome share common intermediate phenotypes on a spectrum of inflammation. Pediatr Crit Care Med.

[14] Kuwata K, Yamada S, Kinuwaki E, Naito M, Mitsuya H (2006). Peripheral hemophagocytosis: an early indicator of advanced systemic inflammatory response syndrome/hemophagocytic syndrome. Shock.

[15] Karras A, Hermine O (2002). Syndrome d’activation macrophagique. Rev Médecine Interne.

[16] John TM, Jacob CN, Ittycheria CC (2012). Macrophage activation syndrome following Acinetobacter baumannii sepsis. Int J Infect Dis.

[17] Buyse S, Teixeira L, Galicier L (2010). Critical care management of patients with hemophagocytic lymphohistiocytosis. Intensive Care Med.

[18] Lin TF, Ferlic-Stark LL, Allen CE, Kozinetz CA, McClain KL (2011). Rate of decline of ferritin in patients with hemophagocytic lymphohistiocytosis as a prognostic variable for mortality. Pediatr Blood Cancer.

